# Neuroprotective Effect of Inosine Against Rotenone Induced Motor deficit & Biochemical Abnormalities in Wistar Rats

**DOI:** 10.1002/alz.085815

**Published:** 2025-01-03

**Authors:** Manmohan Singhal, Mohit Agrawal, Hema Chaudhary

**Affiliations:** ^1^ Faculty of Pharmacy, School of Pharmaceutical & Population Health Informatics, DIT University, Dehradun, Uttrakhand India; ^2^ School of Medical & Allied Sciences, K.R. Mangalam University, Gurugram, Haryana India

## Abstract

**Background:**

Parkinson’s disease is a hypokinetic disorder characterized by selective loss of dopaminergic in substantia nigra pars compacta (SNPc) region of mid‐brain. Dopaminergic degeneration of neurons is considered to be due to oxidative stress, neuroinflammation, neurons mitochondrial dysfunction and glutamate excitotoxicity etc. Inosine a purine nucleoside has been reported to produce anti‐oxidant, anti‐inflammatory and neuromodulatory actions in previous studies. In the current study we have investigated the role of Inosine against rotenone induced motor deficit and biochemical abnormalities in Wistar rats

**Method:**

Rats were treated with 1.5mg/kg rotenone subcutaneously for 35 days. Inosine (50,100 and 200 mg/kg i.p.) was administered after 30 minutes of rotenone administration from day 7^th^ to 35^th^ day. Rotenone caused significant reduction in motor functions and body weight and produced elevation in striatal oxidative burden. The biochemical analysis revealed that there is reduction in the level of anti‐oxidants (GSH, Catalase, SOD) in the animals following rotenone administration. Whereas inosine treated rats were stable and regained their body weight.

**Result:**

In addition inosine significantly attenuated rotenone induced motor deficit and striatal oxidative stress. Outcomes of the current study suggest neuroprotective potential of inosine and its ability to correct movement disability and thus could prone to be useful candidate molecule in the management of motor disorders.

**Conclusion:**

Outcomes of the current study suggest neuroprotective potential of Inosine and its ability to correct movement disability and thus could prove to be useful candidate molecule in the management of motor disorders.

